# Holographic leaky-wave metasurfaces for dual-sensor imaging

**DOI:** 10.1038/srep18170

**Published:** 2015-12-10

**Authors:** Yun Bo Li, Lian Lin Li, Ben Geng Cai, Qiang Cheng, Tie Jun Cui

**Affiliations:** 1 State Key Laboratory of Millimeter Waves, Southeast University, Nanjing 210096, China; 2 Department of Electronics Engineering and Computer Science, Peking University, Beijing, 100871, China; 3 Synergetic Innovation Center of Wireless Communication Technology, Southeast University, Nanjing, 210096, China

## Abstract

Metasurfaces have huge potentials to develop new type imaging systems due to their abilities of controlling electromagnetic waves. Here, we propose a new method for dual-sensor imaging based on cross-like holographic leaky-wave metasurfaces which are composed of hybrid isotropic and anisotropic surface impedance textures. The holographic leaky-wave radiations are generated by special impedance modulations of surface waves excited by the sensor ports. For one independent sensor, the main leaky-wave radiation beam can be scanned by frequency in one-dimensional space, while the frequency scanning in the orthogonal spatial dimension is accomplished by the other sensor. Thus, for a probed object, the imaging plane can be illuminated adequately to obtain the two-dimensional backward scattered fields by the dual-sensor for reconstructing the object. The relativity of beams under different frequencies is very low due to the frequency-scanning beam performance rather than the random beam radiations operated by frequency, and the multi-illuminations with low relativity are very appropriate for multi-mode imaging method with high resolution and anti- noise. Good reconstruction results are given to validate the proposed imaging method.

Due to the low loss, low cost, and high performance, metasurfaces have found huge potential applications in controlling the electromagnetic waves in recent years, which are considered as the two-dimensional (2D) planar metamaterials^1^,^2^,^3^,^4^. The extraction of metasurface’s characteristic parameter is quite different from that of metamaterial. The method of generalized sheet transition condition^5^,^6^ has been used to calculate the spatial reflection and transmission coefficients of metasurfaces by introducing the concept of electric and magnetic polarizability densities. For surface waves propagating on metasurfaces, the transverse resonance approach^7^ was applied to obtain the surface impedance or surface refractive index. Recently, the generalized Snell’s law has been proposed by introducing the concept of phase abrupt^8^ on the metasurface, and sequentially, many excellent works on shaping wavefronts using single-layer sheets have been presented^9^,^10^,^11^,^12^,^13^,^14^,^15^. However, they were still suffered by the limitation of transmission under the cross polarization^16^. Based on the classical electromagnetic theory, the Huygens metasurface was proposed^17^,^18^,^19^ to manipulate the electromagnetic waves without reflections by controlling the electric and magnetic resonances simultaneously.

In the optical frequency, the holographic metasurfaces plays a significant role in shaping the wavefronts and reconstructing the image of an object. Ni *et al*. proposed the thinnest hologram^20^ composed of complementary V-type nano-antennas, which could generate high-resolution and low-noise images by modulating the amplitude and phase. Simultaneously, applying plasmonic metasurfaces, the visual-band image was reconstructed by eliminating undesired influence of multiple diffraction orders^21^. Then, the high conversion efficiency of 80% between two circular polarization states was completed by meta-holograms^22^.

In the microwave band, holographic metasurfaces are frequently used to design antennas in radar and communication systems. In recent years, the metasurfaces shaped by artificial impedance surfaces have many applications in designing leaky-wave antennas^23^. Combining with the microwave holography, the desired radiation waves can be reproduced by exciting the hologram recorded by the interference between the object wave and reference wave^24^,^25^. After that, our group proposed a holographic metasurface that can generate the multi-beam radiations with two-dimensional frequency scanning^26^. The physical equivalence between the leaky waves and holographic antennas based on the Oliner’s method^23^ was given in refs. 26,27. Due to the performance of frequency dispersion, the random holographic leaky-wave metasurfaces for imaging under compressed sensing was recently proposed^28^, which could be considered as the single-sensor imaging system^29^. Then, more practical works^30^,^31^ have been presented to solve the problems of complex-object imaging. However, the random distribution of dispersive unit cells is difficult to design optimally to achieve the requirement of the multi-mode radiations with low relevance for reconstructing the image with high resolution.

In this paper, we propose a new method to realize dual-sensor imaging system using the cross-type holographic leaky-wave metasurfaces which can complete the orthogonally-dimensional beam scanning controlled by frequency. The metasurfaces are composed of the hybrid isotropic and anisotropic metal patches with the grounded dielectric. We use dual-source ports to generate the two-dimensional independent beam scanning under the change of frequency to absolutely illustrate the imaging plane. Then, the multi-frequency backward scattered signals collected by dual sensors can be processed as the method of the conventional single-pixel imaging system^29^. Owing to the directive beam radiations and the distinguished radiation directions by frequencies, good performance of the image reconstruction with high resolution and anti-noise is realized by using the proposed holographic dual-sensor imaging system.

## Results

**Figure 1:**
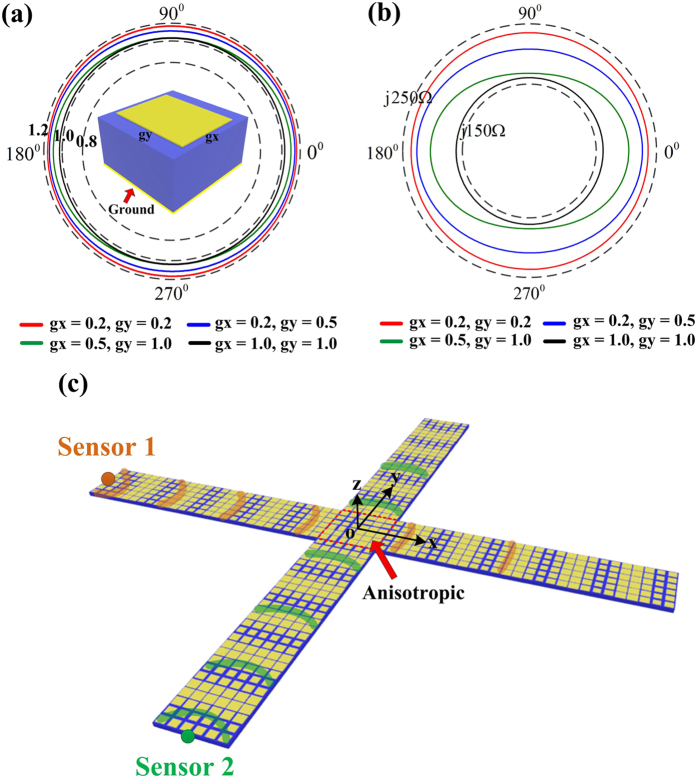
(**a**) The structure of the surface impedance which can realize both isotropic and anisotropic unit cells. We plot the curves of the surface refractive index at 17 GHz by choosing four different textures from our unit database. In the unit database, both 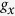 and 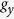 vary from 0.2 to 1.0 mm. (**b**) The relevant curves of the surface impedance. (**c**) The cross-type metasurface for dual-sensor imaging. The anisotropic unit cells are placed in the center and isotropic units are placed on the four arms of the metasurface.

The microwave holographic technology^32^ can generate arbitrary radiation waves by exciting the hologram interference of the reference wave and object wave. If the hologram is expressed as 
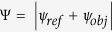
, in which 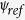 and 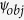 represent the reference wave and object wave respectively, the reproduced wave 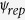 can be given as 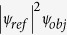 according to the theory of holographic antennas^24^,^27^. We use the gradient surface-impedance textures to shape a cross-type holographic metasurface, as shown in Fig. 1(c). In each arm of the cross, isotropic unit cells are applied; while in the center, anisotropic units are used to satisfy the phase compensating condition of the orthonormal exciting waves. For the exciting source of one direction (e.g., the *x* direction), the distribution of the surface impedance is given as


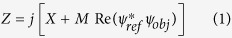


where *X* and *M* represent the average surface impedance and modulation depth, respectively, and “
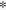
” denotes the conjugate signature. Here, the reference wave 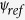 is defined as the cylindrical wave 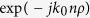, in which 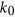 is the wave number in free space, 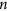 is the effective surface refractive index, and 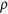 is the distance from a position on metasurface to the original point. 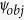 is defined as 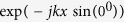, which indicates that the radiation direction of the reproduction wave is zero degree relevant to the normal direction of the metasurface. For the exciting source of another direction (*y* direction), the distribution of surface impedance and the relevant definition are similar to those of the *x* direction. Specifically, on the intersection of the *x* and *y* directions, the surface impedance should simultaneously satisfy the requirement of dual-direction phase matching and hence the anisotropic unit cells are necessary to realize the design.

To implement the isotropic and anisotropic surface impedance unit cells, we choose the basic unit as a subwavelength square (isotropic) or rectangle (anisotropic) metallic patch on a grounded substrate, as shown in Fig. 1(a). The dispersive characteristics of the textures can be obtained by the eigen-mode simulation of commercial software, CST Microwave Studio. For the structures with the grounded dielectric, the transverse-magnetic (TM) mode surface wave is supported^7^,^33^,^34^, and the relevant surface impedance is inductive. For the two-dimensional textures, the effective surface refractive index along the two main axes can be extracted as


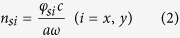


where 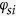 is the surface phase difference across the unit cell along the 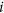 direction, *c* is the light speed in the free space, *a* is the period of the unit cell, and 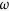 is the angle frequency. The ellipse of the surface refractive index can be described as


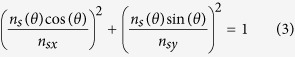


where 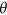 is the propagating direction of surface wave, and 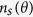 is the corresponding surface refractive index. Thus we can acquire the elliptical surface refractive index curves by changing the texture size, as shown in Fig. 1(a). Due to the TM-mode impedance surface, the surface impedance along the arbitrary propagating direction is then given as


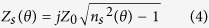


where 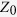 is the impedance in the free space. We plot the surface impedance curves in Fig. 1(b).

According to Eq. (3), we know that if 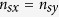, the elliptical curve will become a circle, implying that the impedance texture is isotropic. For 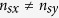, however, the impedance texture can be considered as anisotropic. We can adjust the dual-direction gap sizes between rectangle patches to get the values of the anisotropic surface impedance to satisfy the dual-direction impedance requirement. In our design, on the central area of the metasurface, we can select the appropriate anisotropic unit cells of the least mean square deviation calculated with our anisotropic unit database. For the four arms of the metasurface, the isotropic unit cells are easier to design. We should only establish the relationship between the gap size and the value of the isotropic surface impedance by polynomial fitting.

**Figure 2:**
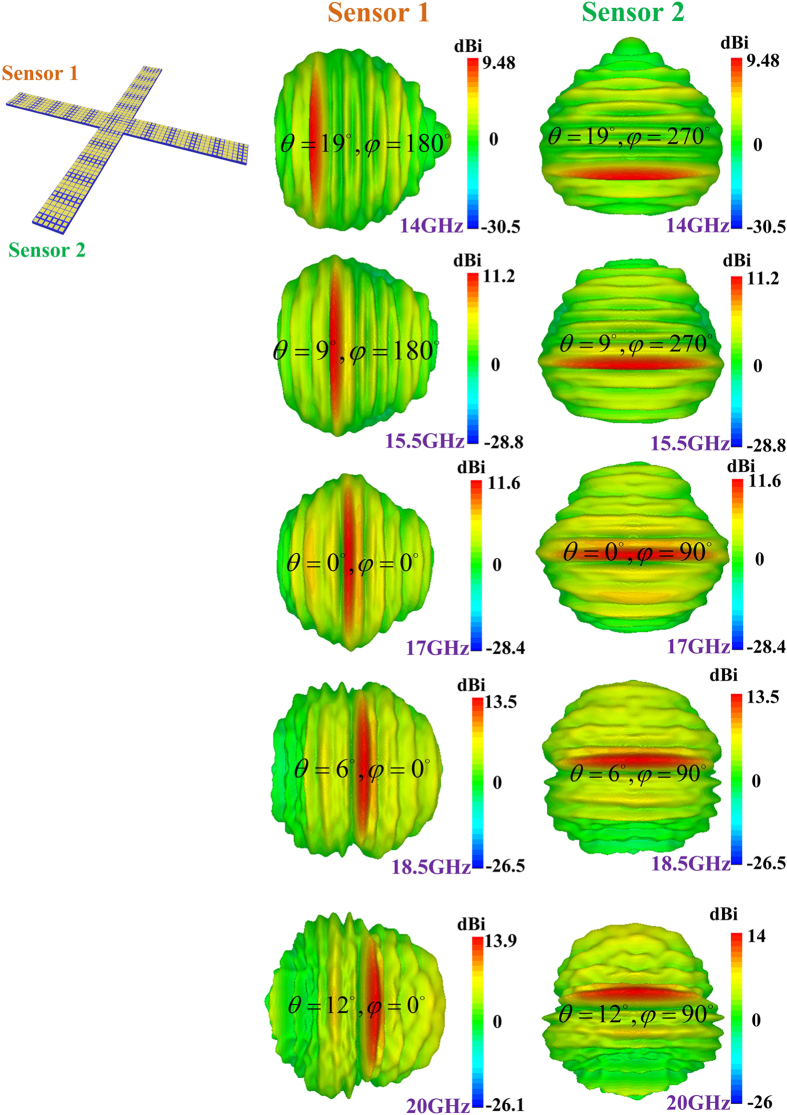
(**a**) The performance of the holographic leaky-wave frequency scanning excited by the dual sensors from 14 to 20 GHz.

In Fig. 1(c), we consider the holographic metasurface as the one-dimensional (1D) period structure corresponding to the *x* or *y* direction. Thus, the regular 1D holographic radiation can be illustrated by the theory of 1D leaky wave under the “−1”-order Floquet’s mode^23^,^26^. If we excite the hologram by the non-coherent (the working frequency is not 17 GHz) reference wave 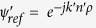 in “sensor 1”, the reproduction wave can be expressed as 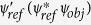, and the phase of which is given as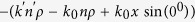, where 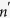 and 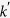 represent the effective surface refractive index and total wave number under the new frequency, respectively. Here, due to the narrow width of the metasurface along the *x* direction, we can assume that 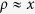. Based on our analysis in ref. 27, the characteristics of the leaky-wave scanning with changing the frequency can be described as


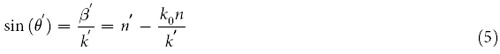


where 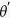
and 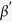
are the radiation direction and surface wavenumber of the reproduction wave under the new frequency, respectively. The performances of the leaky-wave frequency scanning generated by the dual sensors are shown in Fig. 2. We can find that the main scanning beams are very orthonormal, which is beneficial to obtain the high-resolution imaging reconstruction with high noise.

**Figure 3:**
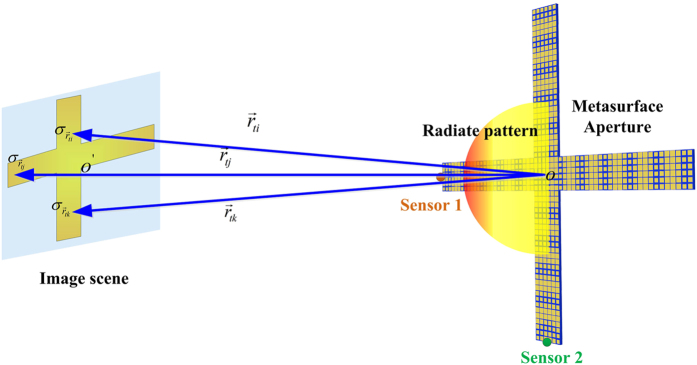
The schematic diagram of the dual-sensor imaging system.

**Figure 4:**
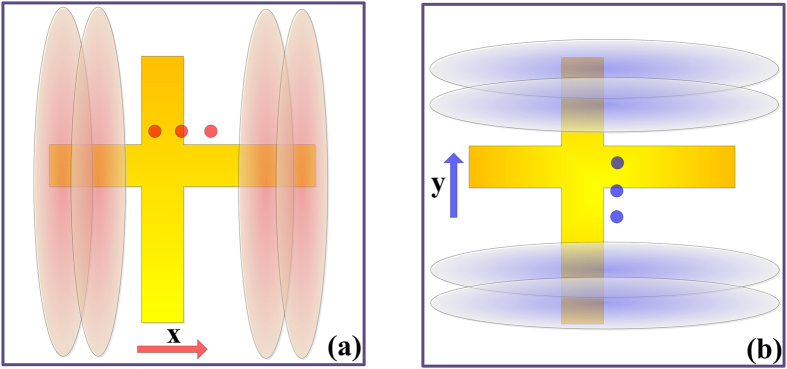
The illustration of the imaging plane. (**a**) The beam scanning along the *x* direction generated by “Sensor 1”. (**b**) The beam scanning along the *y* direction generated by “Sensor 2”.

The schematic diagram of the whole imaging system is demonstrated in Fig. 3. We can independently use each sensor to generate the scanning beams in one dimension to obtain the backward-scattered signals of the object placed in the far field. The scanning beams can widely cover the two-dimensional imaging plane for large field of view (FOV) image under the enough bandwidth of working frequency. If the frequency step corresponding to the step of beam scanning is small, the object plane to be reconstructed can be completely illuminated by the orthonormal scanning beam and the status of the beam illustration is illustrated in Fig. 4.

In Fig. 3, 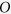 and 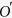 are the original points of the metasurface aperture and imaging plane, respectively, 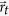 is the transmission vector, and 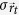 is the radar cross section (RCS) of the sub-object in the imaging plane. In the whole imaging system, the imaging plane is located in the far-field region of the metasurface antenna. As we know, if the object is far enough from the metasurface, the object can be considered as a point source, which is inappropriate for describing the outline of the object. For the proposed dual-sensor imaging system, it has similar signal-processing method to the former single-radar system^28^. In our design, however, the beam can directly illuminate the imaging scene in orthonormal dimensions rather than the random-beam illuminations. To get high-resolution images, low relativity between different beams is necessary and it is very complicated to be realized by optimization under the random design.

According to the theory of single-radar imaging system, we need many different measurement modes, and thus many different metasurface patterns, to constitute a generalized system-response matrix, which will be used to solve the inverse-scattering problem in the single-sensor imaging. Combining with the electromagnetic scattered theory, we obtain the expression of backward scattered signal under one measurement mode as


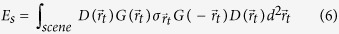


in which, 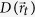 is the radiation pattern generated by the holographic leaky-wave metasurface, 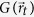
(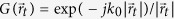) and 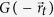 are Green’s functions in three-dimensional free space. The term 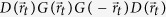 is considered as the system response function, and hence can be written as 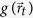 for short. Assuming that the imaging scene is composed of *N* × *N* sub-areas, we can define the sub-RCS of each sub-area as 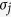
(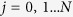). Corresponding to the *j*th sub-area, 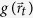 under the *i*th measurement mode can be rewritten as 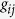
(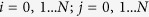). Thus, the *i*th receiving signal under the *i*th measurement mode can be given as 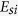
(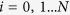):


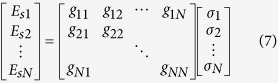


which is further rewritten as 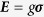 for short. It is obvious that 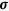 = 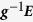 since the matrix ***g*** is a square matrix. When the matrix ***g*** is not a square matrix, the technology of the compressive sensing will be applied to solve the underdetermined matrix equation, which is not the focus of this paper. For our dual-sensor imaging system, the backward signals of the *N*/2 measurement modes will be collected by each sensor. And each mode is generated under the corresponding frequency. Here, in our simulation, *N* = 64, meaning that we need 32 frequency-agile measurement data for each sensor to reconstruct the imaging scene composed of 64 sub-objects. Taking into account the field of view and complete illumination of the imaging scene under the high-gain beams, we choose the working frequencies of the dual-sensor system from 16.25 to 18.5 GHz with the step of 0.07258 GHz. The physical size *L* of the holographic metasurface shown in Fig. 1(c) is 0.18 m. We set the distance from the metasurface aperture to the imaging scene is *R* = 4 m, which satisfies the condition of far fields. The expression of the cross-range resolution in the aperture antenna is given as


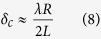


where 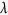 is the wavelength of the central frequency. Hence 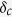 is about 0.1918 m.

**Figure 5:**
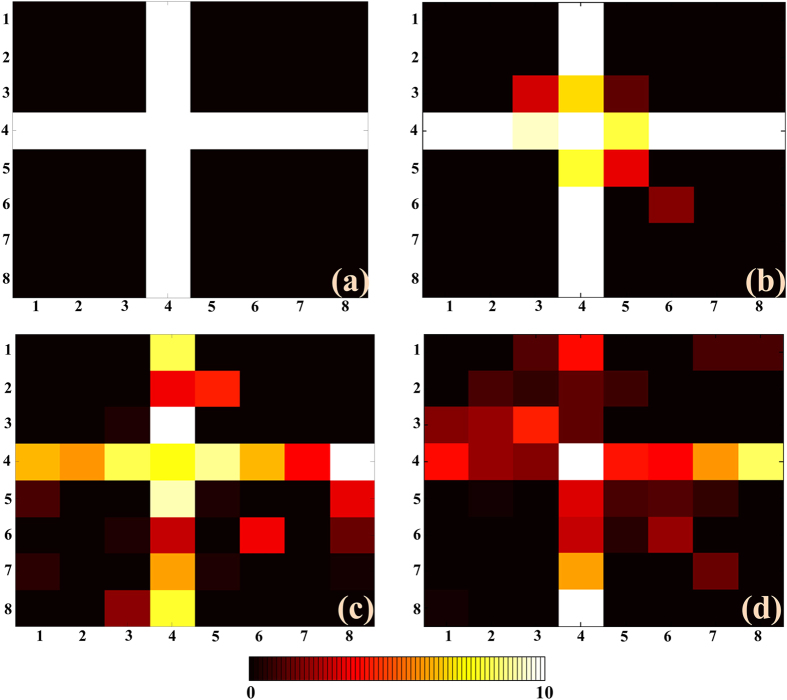
The reconstructed imaging results with the resolution of 0.12 m. The reconstructed images are all composed of 8 × 8 pixels. (**a**) The original object to be reconstructed. (**b–d**) The imaging results under SNRs of 1%, 5%, and 10%, respectively.

In our simulations, we use the full-wave numerical method^35^ to obtain the backward-scattered signals with loading our scanning radiation patterns generated by the metasurface. In the imaging scene, we forwardly add the random noise to the amplitudes and phases of the subareas. Finally, by using the sparse signal processing, the minimum size of the resolution unit, 0.12 m, is acquired in the reconstruction results, as shown in Fig. 5. This resolution is better than the cross-range resolution (Eq. (8)) of the conventional aperture imaging system. In Fig. 5, the cross-type object is reconstructed with adding different-level white noises. Even when the signal-noise ratio (SNR) is 10%, which can be considered as low SNR, the object can still be well reconstructed due to the illumination by our orthonormal radiation patterns.

## Discussion

We proposed a new method of dual-sensor imaging system to obtain high-resolution image reconstructions with the anti-noise performance. The cross-type dual-sensor is made by holographic metasurfaces composed of the hybrid isotropic and anisotropic artificial impedance textures. Combining with the holographic leaky-wave theory, we realized the beam scanning controlled by frequency in the orthonormal dimensions to completely illuminate the imaging scene. The signal-processing method based on the single-radar imaging system is applied in our dual-sensor system. Because the radiation beams are more orthogonal, the matrix equation to solve the inverse problem can tolerate high noise even at the SNR level of 10%. The reconstructed resolution is better than the conventional aperture imaging system. We expect that the dual-sensor imaging system may have the potential applications in the microwave radar imaging and security check.

## Additional Information

**How to cite this article**: Li, Y. B. *et al.* Holographic leaky-wave metasurfaces for dual-sensor imaging. *Sci. Rep.* **5**, 18170; doi: 10.1038/srep18170 (2015).
